# Cordycepin Inhibits the Growth of Hepatocellular Carcinoma by Regulating the Pathway of Aerobic Glycolysis

**DOI:** 10.1155/2022/6454482

**Published:** 2022-11-23

**Authors:** Ya Chen, Yuan Jiang, Xinyu Huang, Lin Chen, Hao Yang, Jian Zhao, Beibei Liang

**Affiliations:** ^1^Shanghai Key Laboratory of Molecular Imaging, Jiading District Central Hospital Affiliated Shanghai University of Medicine and Health Sciences, Shanghai 201318, China; ^2^Shanghai Skin Disease Hospital, School of Medicine, Tongji University, Shanghai 200443, China; ^3^Medical Engineering Department, The Affiliated Hospital of QingDao University, Qingdao 266000, Shandong, China; ^4^Department of Colorectal Surgery, Department of General Surgery, Shanghai East Hospital, Tongji University School of Medicine, Shanghai 200120, China

## Abstract

Hepatocellular carcinoma (HCC) is one of the most common malignant tumors in China, with a high incidence and mortality rate. Glucose metabolism reprogramming is a major characteristic of tumor cells. Increasing evidence indicates that aerobic glycolysis is associated with tumor growth and insensitivity to chemotherapy. Cordycepin inhibits the growth of HCC cells, but the mechanism is yet to be elucidated. Herein, in vitro and in vivo methods were utilized to investigate the cordycepin-inhibited growth of HCC by regulating the metabolic pathway of aerobic glycolysis. In vitro analyses using colony formation and flow cytometry revealed that cordycepin inhibits HCC cells' proliferation and promotes apoptosis. In addition, cordycepin reduced the production of lactic acid and pyruvate, reduced the uptake of glucose, and decreased the extracellular acidification in HCC cells. Specifically, cordycepin inhibited the expression of HK2, LDHA, and PKM2 in aerobic glycolysis via the AMPK-Akt pathway. Taken together, these findings revealed that cordycepin reduces the tumor energy supply and decreases lactic acid production, thereby inhibiting the growth of HCC cells by regulating the metabolic pathway of aerobic glycolysis. These findings might provide novel insights into the mechanisms underlying cordycepin-mediated inhibition of tumor growth as well as a new treatment for HCC.

## 1. Introduction

Liver cancer is a highly malignant tumor [1]. It is classified into four different pathological types: hepatocellular carcinoma (HCC), intrahepatic cholangiocarcinoma (ICC), hepatoblastoma (HB), and mixed liver cancer (HCC-ICC) [2]. The incidence of HCC accounts for 75–85% of liver cancers [3]; however, its pathogenesis is yet to be clarified. Clinical studies demonstrated that in the early stage of cancer, chemotherapeutic drugs can inhibit the growth of cancer cells; however, in the process of tumor development, the migration and invasion of cancer cells weaken the effect of chemotherapeutic drugs [4]. Presently, the treatment of cancer depends on surgical resection, liver transplantation, transcatheter hepatic artery chemoembolization, and radiotherapy, supplemented by targeting, immunity, and chemotherapy [5]. Nonetheless, each of these treatments has its drawbacks. Therefore, the development of new drugs for the treatment of HCC is an urgent requirement [6].

Cordycepin is an antitumor active component, first isolated and purified from the fungus *Cordyceps militaris* by German scientists. Its chemical structure lacks only one hydroxyl group compared to adenosine [7]. Therefore, it can replace adenosine in extracellular regulation, thus inhibiting cell growth. In recent years, cordycepin has played a major role in the treatment of cancer. The underlying mechanisms are summarized as enhancing apoptosis and regulating the cell cycle of tumor cells [8, 9], thereby preventing the metastasis of tumor cells and the synthesis of new substances for cell growth [10, 11], and inhibiting cell proliferation and angiogenesis [12]. Wang et al. identified cordycepin as an effective drug to block radiation ulcers in rats/mice by increasing NRF2 nuclear expression and preventing cell senescence. Cordycepin also activates AMPK by binding to *α*1 and *γ*1 subunits near the self-inhibitory domain of AMPK, promoting p62-dependent Keap1 autophagy degradation, and inducing NRF2 separation from Keap1 and transfer to the nucleus [13]. Although the mechanism of antitumor effects has been reported, the role of cordycepin in inhibiting tumor growth by regulating energy metabolism has not yet been reported.

Adenosine 5-monophosphate-activated protein kinase K (AMPK) regulates intracellular energy metabolism [14]. In higher eukaryotes, AMPK has acquired the ability to sense available energy in cells by directly binding to adenine nucleotides [15]. Once activated, AMPK phosphorylates the key proteins, shifting the metabolism to increased catabolism and reduced anabolism [16]. In addition to direct involvement in the regulation of key enzymes in metabolic pathways, AMPK also reconnects cell metabolism for the long term by targeting transcriptional regulators [17]. Protein kinase *B* (Akt) is a serine/threonine kinase downstream of AMPK, which can be activated by a wide range of growth signals; also, the biochemical mechanism underlying Akt activation is elucidated [18]. Once activated, Akt regulates many downstream proteins related to cell proliferation, migration, and glucose metabolism. Akt is the hub of multiple signaling pathways that are often regulated in various human cancers [19].

Furthermore, for the occurrence and development of cancer, the cells need to reprogram their catabolism and anabolism to obtain sufficient energy and synthetic biomass for cell growth [20]. Warburg et al. demonstrated that even under oxygen-rich conditions, tumor cells metabolize glucose through the glycolysis pathway, i.e., aerobic glycolysis [21]. The rapid metabolism of glucose results in a local anoxic environment outside the tumor cells [22]. Aerobic glycolysis is also considered an adaptive mechanism that supports the metabolic microenvironment of tumors and facilitates rapid biosynthesis. Previous studies have confirmed that phosphofructokinase (PFK), hexokinase 2 (HK2), lactate dehydrogenase *A* (LDHA), and pyruvate kinase 2 (PKM2) are the key proteins in the metabolic pathway of tumor aerobic glycolysis, which are potential targets for tumor drug therapy. The expression of HK2 is upregulated in many cancers [23]. Jian et al. found that in animal models, the absence of HK2 decreased the proliferation of cancer cells without obvious side effects, indicating that targeting HK2 is a feasible cancer treatment strategy. Inhibition of HK2 downregulated aerobic glycolysis, thus affecting various pathways of central metabolism and destabilizing the outer membrane of the mitochondria, resulting in cell death [24].

In summary, the disruption of energy metabolism promotes the proliferation of tumor cells. The Warburg effect is one of the main characteristics of the energy metabolism of tumor cells and a target for cancer treatment. Cordycepin inhibits cancer cell growth; however, the mechanism is not yet clarified. Herein, we hypothesized that cordycepin might inhibit the aerobic glycolysis pathway, thereby inhibiting the production of lactic acid and pyruvate and reducing glucose uptake. The present study confirmed that cordycepin reduces the tumor energy supply and decreases lactic acid production to inhibit HCC growth by regulating the metabolic pathway of aerobic glycolysis. Thus, these findings might provide novel insights into the mechanisms of cordycepin-mediated inhibition of tumor growth.

## 2. Materials and Methods

### 2.1. Materials and Cell Line

Hep-G2 cells (PRID: CVCL_0027) were purchased from Procell Life Science and Technology Co., Ltd. (Wuhan, China); HCC-LM3 cells (PRID: CVCL_4972) were purchased from Hunan Fenghui Biotechnology Co., Ltd. (Wuhan, China). Cordycepin (73-03-0) was obtained from Aladdin (Shanghai, China). All cell lines were tested for mycoplasma contamination. DMEM (KGM12800-500) was purchased from Jiangsu KeyGEN Biotech Corp. (Jiangsu, China). Fetal bovine serum (FBS) was purchased from Absin (abs974; Shanghai, China). HCC cells were treated with 0, 25, 50, or 100 *μ*g/mL of cordycepin for 24 h. 5 FU was used as the positive control. These cells were maintained in DMEM supplemented with 10% (vol/vol) FBS at 37°C and 5% CO_2_.

### 2.2. Cytotoxicity Analysis

The cytotoxicity of cordycepin was confirmed on HepG2 and HCC-LM3 cells using the CCK-8 method. The cells were seeded in 96-well plates at a density of 10000 cells/well and treated with 0, 25, 50, and 100 *μ*g/mL cordycepin, respectively, at 37°C for 24 h, followed by the addition of the CCK-8 reagent (C0038, Beyotime, Shanghai, China). The reaction was incubated with cells for 1 h before measuring the absorbance at 450 nm on a microplate reader (Thermo Fisher Scientific, USA). Subsequently, the tumor cell growth inhibition ratio (TGI) was calculated according to the following formula:

(1) $$\begin{array}{c} \text{TGI}\left(\%\right)=\frac{C-T}{C}\times 100\%, \end{array}$$

where *T* is the average absorbance value of the treated groups and *C* is the average absorbance value of the control group.

### 2.3. Colony Formation Assay

HCC-LM3 and Hep-G2 cells were seeded in 6-well plates at a density of 1000 cells/well and cultured with different concentrations of cordycepin for 14 days. The ensuing colonies were fixed with 4% paraformaldehyde (abs9179, Absin, Shanghai, China) for 30 min at room temperature and stained with 1% crystal violet (G1062, Solarbio, Beijing, China) for 30 min. After rinsing with tap water for 30 min, the colonies (>50 cells) were counted, and the average of three independent experiments was calculated.

### 2.4. Apoptosis Assay

The effect of cordycepin on apoptosis was evaluated using the FITC-Annexin V apoptosis detection kit (556507, BD, USA). Hep-G2 and HCC-LM3 cells were treated with different concentrations of cordycepin for 24 h. Subsequently, the cells were washed with cold phosphate-buffered saline (PBS) and resuspended in 500 *μ*L of 1× binding buffer. An equivalent of 100 *μ*L cell suspension was mixed with 5 *μ*L Annexin V-FITC and 5 *μ*L PI and incubated at 25°C for 15 min in the dark. Then, 400 *μ*L of 1× binding buffer was added, and the cells were analyzed by flow cytometry (Agilent, USA). The data were shown as the percentage of the cells represented by alive, apoptosis, and population.

### 2.5. Lactate Production Assays

HCC-LM3 and Hep-G2 cells were seeded onto 6-well plates at a density of 1 × 10^6^ cells/well and treated with cordycepin at 50 *μ*g/mL and 100 *μ*g/mL for 24 h. Subsequently, the culture supernatant was collected by centrifugation at 1000 RPM for 5 mins, and the cell pellets were resuspended in 700 *μ*L DMEM medium for 12 h. Then, the lactate concentrations in cell lysates were detected using the Lactate Assay Kit (A019-2-1, NanJing JianCheng Bioengineering Institute, Nanjing, China), according to the protocol. The absorbance was measured at 530 nm on a microplate reader.

### 2.6. Glucose Uptake Assays

Glucose uptake was detected using the glucose (GO) assay kit (GAGO20, Sigma–Aldrich, St. Louis, MO, USA). HCC-LM3 and Hep-G2 cells were plated in 6-well plates at a density of 1 × 10^6^ cells/well and cultured with cordycepin at different concentrations for 24 h. Then, the cells were washed with PBS and cultured in glucose-free DMEM for 6 h. Subsequently, the supernatants were collected to measure the glucose uptake, following the manufacturer's instructions. The glucose consumption was calculated by deducting the measured glucose concentration in the medium from the original glucose concentration.

### 2.7. Pyruvate Kinase Activity

Pyruvate kinase activity was detected using the kit (Solarbio). HCC-LM3 and Hep-G2 cells were plated at a density of 1 × 10^6^ cells/well in 6-well plates and treated with different concentrations of cordycepin for 24 h. Subsequently, the cells were counted in the precipitate, and the supernatant collected by centrifugation was transferred to a 1.5 mL tube. All estimations were performed according to the manufacturer's instructions.

### 2.8. Extracellular Acidification Rate (ECAR)

Cellular oxidative phosphorylation and glycolysis alterations were determined on a Seahorse XF24 Flux Analyzer (Seahorse Bioscience, Agilent, USA) by measuring the ECARs in real-time according to the manufacturer's instructions. The HCC-LM3 and Hep-G2 cells treated with cordycepin at 50 *μ*g/mL and 100 *μ*g/mL for 24 h were seeded on a XF24-well plate at a density of 350,000 cells/well overnight in the incubator. On the following day, the culture medium was removed from the well; the cells were washed and replaced with 500 *μ*L hippocampal solution (nonbuffer system DMEM), 37°C, without CO_2_ incubator for 30 min–1 h. Glucose, oligomycin, and 2-deoxyglucose were added sequentially to the four sample wells, such that the final working concentrations were 10 mM, 1 *μ*M, and 50 mM. The 24-well plate was placed in the hippocampal instrument for calibration (about 20–30 mins), following which the plate was subjected to the dynamic detection of the acidification rate of the culture medium around the cells. The ECAR measurements were normalized to cell numbers and reported as mpH/min.

### 2.9. RNA Preparation and Fluorescence Real-Time Quantitative Polymerase Chain Reaction (RT-qPCR)

Total RNA was isolated from the cells using the EZgene Tissue RNA Miniprep Kit (Biomiga, USA), according to the manufacturer's instructions. An equivalent of 500 ng of RNA was reverse transcribed into cDNA using HiScript RT SuperMix (Vazyme, NanJing, China) and used as a template for RT-qPCR, performed using ChamQ™ SYBR Color qPCR Master Mix (Vazyme, NanJing, China). The primer sequences are listed in Supplementary Table 2. The experiments were repeated at least three times.

### 2.10. Western Blot Assays

HCC-LM3 and Hep-G2 cells were extracted from a 6-well plate, collected by centrifugation, and lysed in 200 *μ*L lysis buffer (KeyGEN, Jiangsu, China) containing 1% PMSF (KeyGEN, Jiangsu, China) on ice. Subsequently, the supernatants were collected by centrifugation at 15000 × *g*, 4°C for 5 mins. The protein concentrations were measured using the Bicinchoninic Acid (BCA) kit (YEASEN, Shanghai, China). An equivalent of 20 *μ*g protein sample was resolved by SDS-PAGE and transferred to a PVDF membrane (Immobilon, Ireland), blocked with 5% nonfat milk (EpiZyme, Shanghai, China), and probed with primary antibodies at 4°C. Akt Rabbit antibody (Ab) (9272S), p-Akt Rabbit mAb (4058S), AMPK*α* antibody (2532S), p-AMPK*α* (T172) Rabbit Ab (2531S), PKM2 (4053S), and LDHA (3582S) at 1 : 1000 (Cell Signaling Technology). Bcl-2 rabbit polyclonal antibody (AF6285) and Caspase-3 (active) Rabbit mAb (AC033) were used at 1 : 1000 (Beyotime). Hexokinase2 polyclonal antibody (22029-1-AP) at 1 : 1000 was purchased from Proteintech. After incubation with the secondary antibody for 1 h at RT, the immunoreactive bands were detected by enzyme-linked chemiluminescence (ECL) (Yeasen, Shanghai, China) and quantified using a Chemiluminescent and Fluorescent Imaging System (Tanon, China). Horseradish peroxidase (HRP)-labeled goat anti-mouse IgG (LF101) was used at 1 : 10000 (EpiZyme). HRP-labeled goat anti-rabbit (A0208) was used at 1 : 10000 (Beyotime). The Antibodies are listed in Supplementary Table 1.

### 2.11. Animal Studies and Images

Male athymic BALB/c nude mice, aged 4 weeks, were purchased from the Shanghai Experimental Animal Center of the Chinese Academy of Sciences (Shanghai, China). The mice protocols were in accordance with the guidelines established by the National Institutes of Health Guide for the Care and Use of Laboratory Animals. HCC-LM3 cells were cultured until the cell density reached 5 × 10^7^/mL. Then, 1 × 10^7^ cells were inoculated in a 200 *μ*L volume on the right back of the nude mice. Once the tumors reached an average volume of 100 mm^3^, the mice were randomly divided into five groups (each group, *n* = 5) and treated as follows: group I: PBS as the control; group II: 10 mg/kg cordycepin; group III: 20 mg/kg cordycepin; group IV: 10 mg/kg 5 FU; and group V: 10 mg/kg cordycepin + 10 mg/kg 5 FU. The intraperitoneal injection was administered once a day. The tumor size was measured using Vernier calipers every 3 days, and the volume was calculated using the formula: 0.5 × (Length × Width^2^), wherein *L* represents the long diameter, and *W* represents short diameter. After 30 days, the mice were sacrificed.

### 2.12. ^18^F FDG-PET/CT

Before imaging, the rats were fasted for 6–8 h and weighed. About 5 mBq ^18^F-FDG developer was injected into nude mice by caudal vein injection. After 40 min, 2% isoflurane gas was inhaled, and after 5 min, whole-body PET/CT (Mediso, Hungary; Bioscan, USA) scanning was conducted. Subsequently, PET images were acquired on the computer. The PET images of nude mice were captured on the workstation, and SUVmax was measured (continuously at five tumor levels, and the mean value was the final SUVmax of the tumor).

### 2.13. Immunohistochemical (IHC) Staining

The paraffin sections were sliced into 3–5 mm thin slices on a tissue slide and baked at 65°C for 1 h to prevent peeling. The baked slices were placed in xylene for 10 mins, followed by 95% ethanol and 75% ethanol for 5 min each. Then, the slices were rinsed in double-distilled water, immersed in PBS buffer (pH 7.2), and soaked in a 3% hydrogen peroxide-methanol solution (90 mL methanol/10 mL 30% hydrogen peroxide solution) at room temperature for 15 mins to eliminate the effects of endogenous peroxidase. After PBS washes, the slices were sealed in a humid chamber with 20% FBS for 30 mins. For antigen retrieval, the slides were heated in citrate buffer. Then, the slides were incubated with primary antibodies against PKM2 (1 : 800; 4053S; Cell Signaling Technology), HK2 (1 : 50, 22029-1-AP, Proteintech), and LDHA (1 : 800; 3582S; Cell Signaling Technology) at 4°C, overnight, followed by incubation with secondary antibody at 37°C for 1 h. After DAB color development (Dako, Carpinteria, CA, USA), the slides were stained with hematoxylin (Riblology, Shanghai, China) for 15 s, washed under tap water, dried at 65°C, and sealed with a coverslip and neutral gum before being examined under the microscope.

### 2.14. TUNEL. Dewaxing: Same as IHC Staining

The TUNEL staining kit was purchased from Beyotime. The wet sections were incubated with protease K solution at 37°C for 10–30 mins, followed by PBS washes. Then, the slides were dried before adding 50 *μ*L TDT enzyme working solution, and the reaction was facilitated at 37°C for 60 mins in the dark. Subsequently, 50 *μ*L of streptavidin-HRP was added to the slides and incubated at 37°C for 30 mins in the dark. Finally, a fluorescence quenching and sealing solution was used to seal the slides before observing them under a fluorescence microscope.

### 2.15. Statistical Analysis

The group results were expressed as the mean ± standard deviation of at least three repeated samples. The differences between groups were analyzed using GraphPad Prism and SPSS statistical software (SPSS, Chicago, IL, USA). *P* < 0.05 indicated statistically significant results.

## 3. Results

### 3.1. Cordycepin Inhibits the Proliferation of HCC Cells

**Figure 1:**
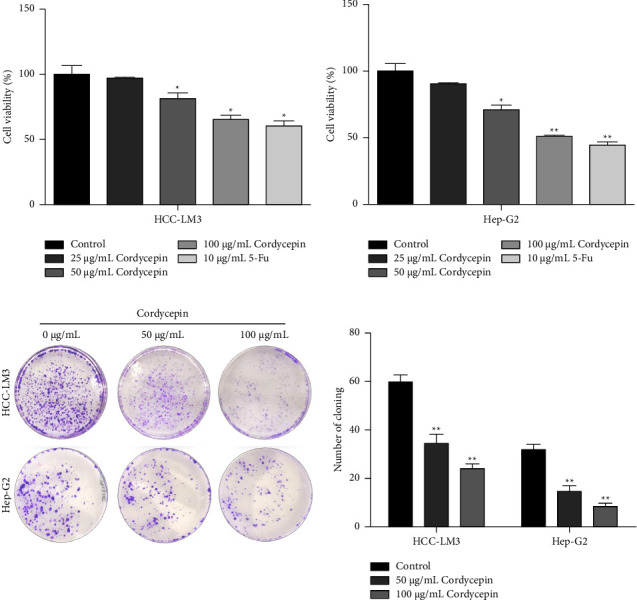
Cordycepin inhibits the proliferation of HCC cells. (a, b) The effect of cordycepin on the proliferation of HCC-LM3 and Hep-G2 cells was detected by CCK-8 assay. HCC cells were exposed to 0, 25, 50, and 100 *μ*g/mL cordycepin and 10 *μ*g/mL 5 FU for 24 h. (c) The effect of cordycepin on colony formation ability of HCC-LM3 and Hep-G2 cells. HCC-LM3 and Hep-G2 cells were treated with 0, 50, and 100 *μ*g/mL cordycepin. Data are presented as means ± SD from triplicate experiments. Statistical significance was indicated as ^∗^*P* < 0.05 and ^*∗∗*^*P* < 0.01 vs. control group.

To detect the effects of cordycepin on tumor cell proliferation, CCK-8 assays and plate cloning experiments were performed on HCC-LM3 and Hep-G2 cells. The CCK-8 assays showed that cordycepin inhibits the proliferation of HCC-LM3 and Hep-G2 cells after treatment with 25, 50, and 100 cordycepin and 10 *μ*g/mL 5 FU as a positive control for 24 h, and the cell proliferation was significantly decreased with the increase in cordycepin concentration (Figures 1(a) and 1(b)). Next, we detected the effects of cordycepin on the colony formation of HCC-LM3 and Hep-G2. Compared to the control group, the size of the colonies in the cordycepin treatment group was smaller, and their number decreased significantly (Figure 1(c)).

### 3.2. Cordycepin Induces Apoptosis in HCC Cells

**Figure 2:**
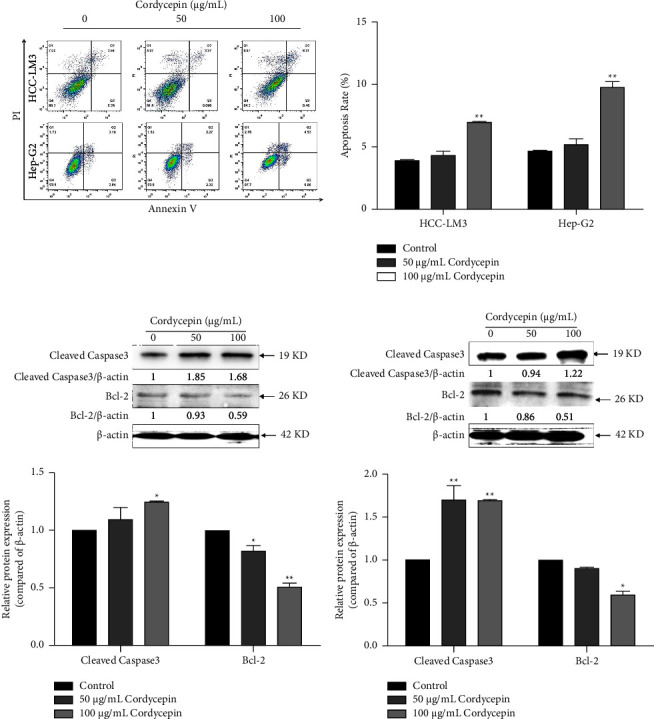
Cordycepin induces apoptosis in HCC-LM3 and Hep-G2. (a) 100 *μ*g/mL cordycepin induces apoptosis in HCC-LM3 and Hep-G2. The apoptotic rate of HCC-LM3 and Hep-G2 cells was detected by flow cytometry. The effect of cordycepin on the expression of apoptosis-related protein in HCC-LM3 (b) and Hep-G2 (c) cells was detected by Western blotting. HCC-LM3 and Hep-G2 cells were treated with 0, 50, and 100 *μ*g/mL cordycepin. Data are expressed as means ± SD. ^∗^*P* < 0.05; ^*∗∗*^*P* < 0.01 (vs. control group, *n* = 3).

Apoptosis is a form of programmed cell death. Apoptotic, live, and necrotic cells can be distinguished using AnnexinV/PI double staining. HCC-LM3 and Hep-G2 cells were treated with different concentrations of cordycepin for 48 h. The apoptotic rate of HCC-LM3 and Hep-G2 cells was detected by flow cytometry. The results showed that, compared to the control group, 50 *μ*g/mL cordycepin had no significant effect on HCC cells (^*∗*^*P* > 0.05), while 100 *μ*g/mL cordycepin induced apoptosis in HCC-LM3 cells. The apoptotic rate of Hep-G2 cells was 10.7%, which was significantly higher than that of the control group (^*∗∗*^*P* < 0.01) (Figure 2(a)). The effects of cordycepin on apoptosis-related proteins in HCC-LM3 and Hep-G2 cells were detected by Western blot assay. Then, HCC-LM3 and Hep-G2 cells were treated with 50 *μ*g/mL and 100 *μ*g/mL cordycepin for 24 h, and the expression of apoptotic proteins Bcl-2 and cleaved caspase3 was detected by Western blotting. The results showed that the expression of Bcl-2 in HCC-LM3 and Hep-G2 was downregulated and that of the cleaved caspase3 protein was upregulated after 100 *μ*g/mL cordycepin treatment (Figures 2(b) and 2(c), ^*∗*^*P* > 0.05 or ^*∗∗*^*P* < 0.01). These findings confirmed that cordycepin induces apoptosis in HCC-LM3 and Hep-G2 cells.

Autophagy is another form of programmed cell death. We would make sure whether cordycepin-induced cell death could result from autophagy. So, we measured and compared the expression of autophagy-regulated target molecules and autophagosomes. The results showed no significant difference between the control and cordycepin groups in the expression of ATG5, Beclin1, or LC3 in HCC-LM3, Hep-G2 cells, and HCC-LM3 tumor-bearing nude mice tissues (Supplementary Figures 1(a), 1(b), 1(d), and 1(e)). In addition, the number of autophagosomes could not be induced obviously after 100 *μ*g/mL cordycepin treatment in Hep-G2 compared with the control group, as determined by transmission electron microscopy (Supplementary Figure 1(c)). All these results confirmed that Cordycepin does not induce the autophagy cell death pathway.

### 3.3. Cordycepin Regulates Aerobic Glycolysis in HCC Cells

**Figure 3:**
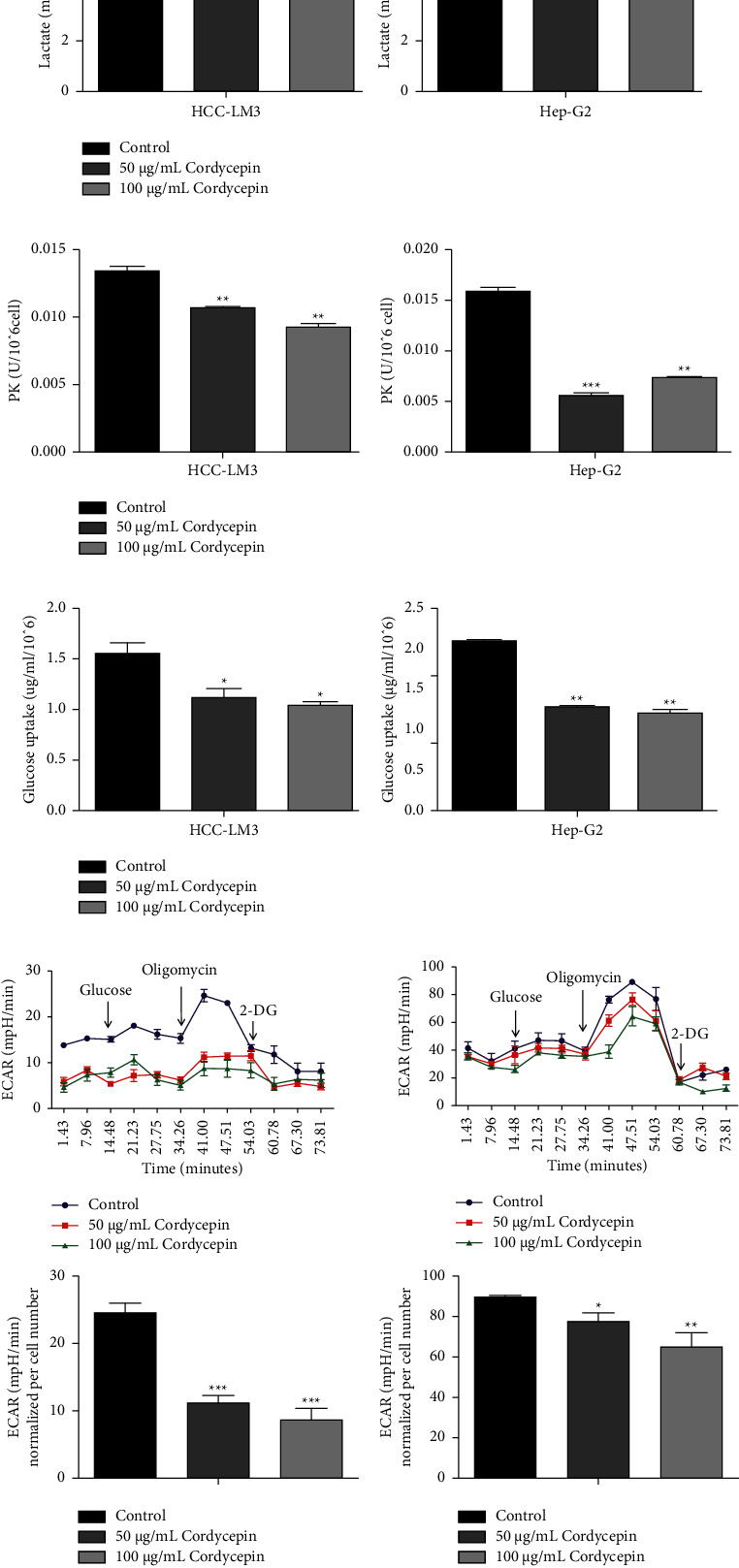
Cordycepin affects the metabolic process of aerobic glycolysis. (a) Effect of cordycepin on lactic acid secretion. Cordycepin inhibits the secretion of lactic acid after 24 h of treatment. (b) Effect of cordycepin on pyruvate kinase secretion. Cordycepin inhibits the secretion of pyruvate kinase after 24 h treatment. (c) Glucose uptake was suppressed by cordycepin treatment at different concentrations. HCC-LM3 (d) and Hep-G2 (e) cells were treated with different concentrations of cordycepin for 12 h. ECAR was detected by the seahorse XF24 extracellular flux analyzer using the glycolysis stress test kit. Cordycepin treatment decreased ECAR in HCC-LM3 and Hep-G2 cells. Data are expressed as means ± SD. ^∗^*P* < 0.05; ^*∗∗*^*P* < 0.01; ^*∗∗∗*^*P* < 0.001 (vs. control group, *n* = 3).

Lactic acid is the primary end-product of glucose metabolism, and a proportion is excreted out of the cell. The metabolic level of aerobic glycolysis is indirectly reflected by estimating the content of the extracellular lactic acid. After Hep-G2 and HCC-LM3 cells were treated with 50 *μ*g/mL and 100 *μ*g/mL cordycepin, the lactic acid content was detected using a lactic acid detection kit. The results showed that the extracellular lactic acid production of HCC-LM3 and Hep-G2 cells treated with 50 *μ*g/mL and 100 *μ*g/mL cordycepin was significantly decreased compared to the controls. This phenomenon suggested that cordycepin limits the production of lactic acid in the metabolic process of aerobic glycolysis in HCC cells (Figure 3(a)).

Pyruvate is the intermediate metabolite of the glycolysis pathway, and a proportion of pyruvate can be excreted from the cell while some content is retained for the subsequent enzymatic reaction to produce lactic acid. After treating Hep-G2 and HCC-LM3 cells with 50 and 100 *μ*g/mL of cordycepin for 24 h, the cells were lysed, and the pyruvate content was estimated using a pyruvate detection kit. The results showed that the content of pyruvate in Hep-G2 and HCC-LM3 cells treated with 50 *μ*g/mL and 100 *μ*g/mL cordycepin was significantly lower than that in control cells (^*∗∗∗*^*P* < 0.001). This phenomenon suggests that cordycepin decreases the production of pyruvate in the metabolic process of aerobic glycolysis in HCC cells (Figure 3(b)).

After Hep-G2 and HCC-LM3 were treated with 50 *μ*g/mL and 100 *μ*g/mL cordycepin, the glucose content in the culture medium of each group was measured using a glucose detection kit. The results showed that, compared to the control group, the glucose uptake of HCC-LM3 and Hep-G2 cells treated with 50 *μ*g/mL and 100 *μ*g/mL cordycepin was significantly decreased, indicating that cordycepin inhibits glucose uptake in HCC cells (Figure 3(c)).

The glucose metabolism produces lactic acid, which leads to the production and excretion of protons in the extracellular culture medium. In this study, the acidity of the culture medium was directly detected by the hippocampal XF24 extracellular flux analyzer, i.e., the ECAR. The extracellular acidification of hepatoma cells (LM3 and Hep-G2) in each group was assessed on XF24 at 50 *μ*g/mL and 100 *μ*g/mL cordycepin. The results showed that, compared to the control group, the ECAR of the 50 and 100 *μ*g/mL cordycepin treatment groups significantly was decreased (Figures 3(d) and 3(e)). This suggests that cordycepin inhibits the ECAR rate of HCC cells.

### 3.4. Cordycepin Regulates the Aerobic Glycolysis Pathway through the AMPK-Akt-HK2/PKM2/LDHA Axis

**Figure 4:**
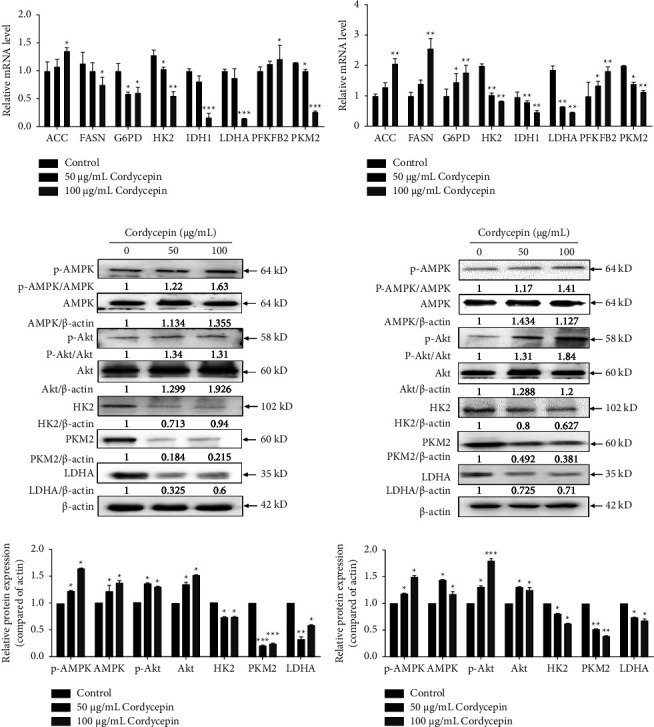
Cordycepin regulates the aerobic glycolysis pathway through the AMPK/Akt-HK2/PKM2/LDHA axis. The expression of key target genes in glucose and lipid metabolism in HCC-LM3 (a) and Hep-G2 (b) cells treated with 0, 50, and 100 *μ*g/mL cordycepin for 24 h by RT-qPCR assay. The expression of phosphorylated AMPK/Akt and the key target proteins of aerobic glycolysis (HK2, PKM2, and LDHA) were detected by Western blot in HCC-LM3 (c) and Hep-G2 (d) cells treated with cordycepin 0, 50, and 100 *μ*g/mL for 24 h. Data are expressed as means ± SD. ^∗^*P* < 0.05; ^*∗∗*^*P* < 0.01; ^*∗∗∗*^*P* < 0.001 (vs. control group, *n* = 3).

In order to further explore the mechanism of cordycepin on aerobic glycolysis, Hep-G2 and HCC-LM3 cells, treated with 50 *μ*g/mL and 100 *μ*g/mL cordycepin for 24 h, were selected, and the key target genes related to glucose metabolism and lipid metabolism were detected by RT-qPCR assay. The results confirmed that the mRNA levels of *HK2*, *PKM2*, and *LDHA* were significantly reduced in HCC-LM3 and Hep-G2 cells after treatment with 50 *μ*g/mL and 100 *μ*g/mL cordycepin (Figures 4(a) and 4(b)).

Furthermore, the upstream regulatory targets of cordycepin regulating the metabolic pathway of aerobic glycolysis were investigated. Thus, Hep-G2 and HCC-LM3 cells were treated with 50 *μ*g/mL and 100 *μ*g/mL cordycepin, respectively. The expression of AMPK, p-AMPK, Akt, p-Akt, HK2, PKM2, and LDHA, the key targets of aerobic glycolysis, was detected in these cells. The expression of p-AMPK and p-Akt was upregulated, and key enzymes of aerobic glycolysis (HK2, PKM2, and LDHA) were significantly downregulated in the cordycepin groups compared to the control groups (Figures 4(c) and 4(d)). We concluded that cordycepin regulates the aerobic glycolysis pathway through the AMPK-Akt-HK2/PKM2/LDHA axis in HCC.

### 3.5. Cordycepin Suppresses Tumor Growth In Vivo by Regulating Aerobic Glycolysis

**Figure 5:**
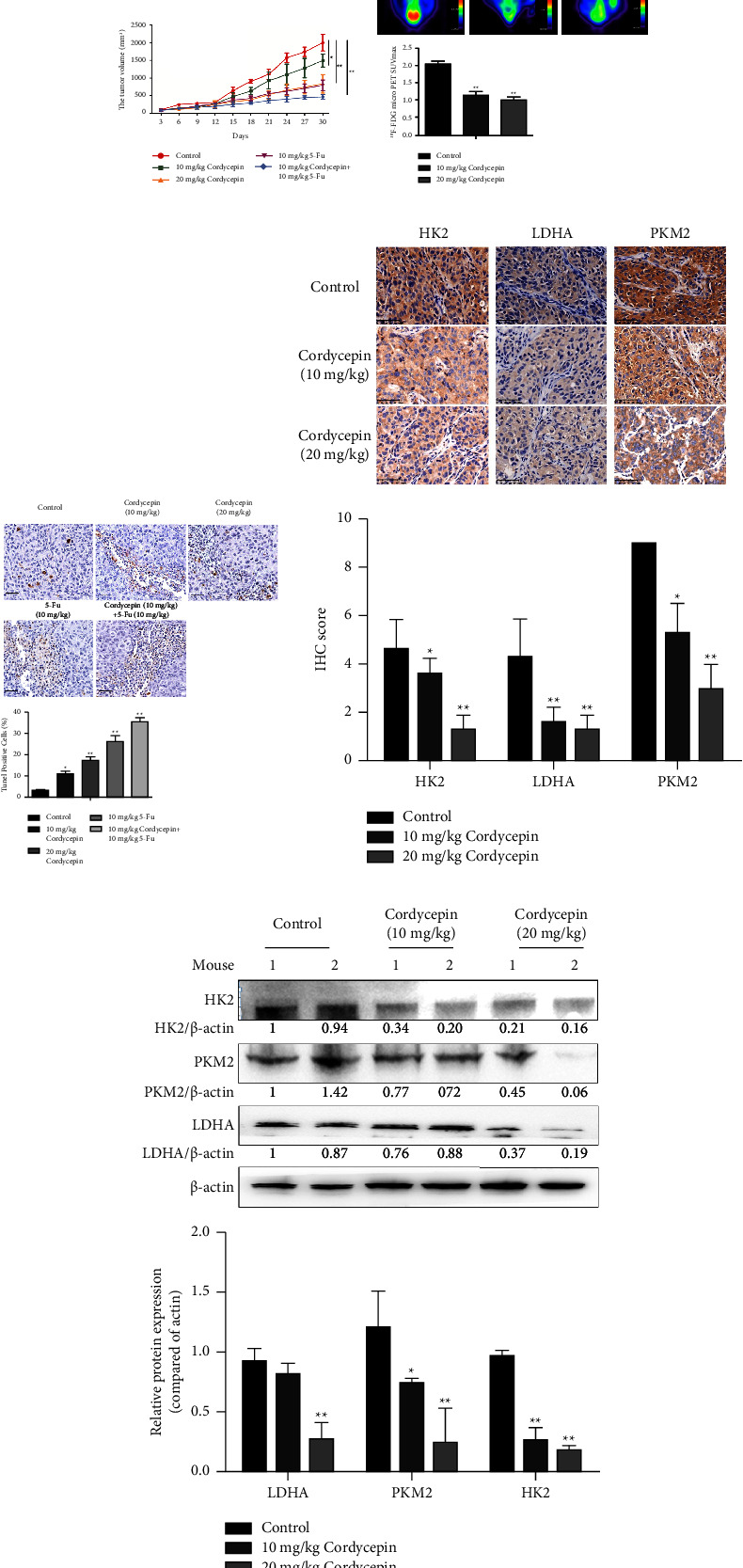
Cordycepin suppresses tumor growth in the tumor xenograft model. (a) Observation of the tumor mass morphology of transplanted tumors in nude mice in various medication groups. Cultured cells were inoculated in nude mice at 1 × 10^7^ cells/mL and randomly divided into groups after 10 days: PBS, 10 *μ*g/mL cordycepin, 20 *μ*g/mL cordycepin, 10 *μ*g/mL 5 FU, and 10 *μ*g/mL cordycepin + 10 *μ*g/mL 5 FU. Intraperitoneal injection was administered once a day, and the combination group was given drugs at daily intervals for 30 days. The tumor volume of nude mice was measured after 3 days. The statistical data showed tumor volume between different treatment groups. (b) ^18^F-FDG micro-PET imaging and statistical analysis of tumors in different drug groups. Experimental data showed a glucose uptake in nude mouse tumor tissues in different groups and indirectly reflected the glucose metabolism level; the white arrow indicates the location of the xenograft tumor. (c) TUNEL staining (magnification ×200) of tumor tissues of tumor-bearing nude mice in different drug groups and statistical analysis. (d) IHC staining and statistical analysis of tumor tissues in HCC-LM3 tumor-bearing nude mice and quantification of the expression of HK2, LDHA, and PKM2 molecules. (e) Expression levels of HK2, PKM2, and LDHA proteins in tumor tissues of HCC-LM3 tumor-bearing nude mice. Data are expressed as mean ± SD. ^∗^*P* < 0.05; ^*∗∗*^*P* < 0.01; ^*∗∗∗*^*P* < 0.001 (vs. control group, *n* = 3).

**Figure 6:**
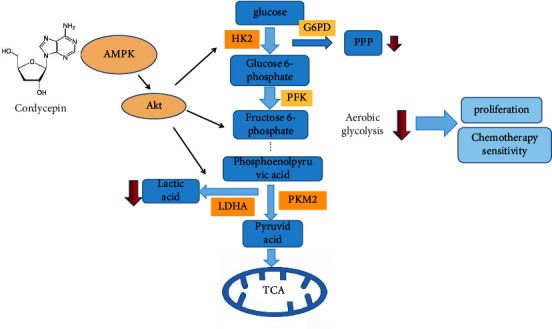
Schematic representation of cordycepin-inhibited tumor growth via aerobic glycolysis. Cordycepin inhibits the growth of HCC cells and promotes tumor chemotherapy sensitivity by regulating the AMPK/Akt-HK2/PKM2/LDHA axis in HCC.

The transplanted tumor model of HCC-LM3 tumor-bearing nude mice was established and randomly divided into five groups (the control group, the 10 mg/kg cordycepin group, the 20 mg/kg cordycepin group, the 10 mg/kg 5 FU group, the 10 mg/kg cordycepin group, and the 10 mg/kg 5 FU group) after the tumor grew up to day 10 (*n* = 5 nude mice/group). 5 FU is one of the most commonly used chemotherapeutic agents for gastric cancer. 5 FU has a direct cytotoxic effect on tumors, and drug combinations are a commonly used clinical treatment for tumors. In this study, we designed a combined administration group. The tumor volume and analysis of nude mice in the different treatment groups are shown in Figure 5(a). We found that the combination group was more effective than cordycepin or 5 FU alone. The results showed that the tumor volume in the 10 mg/kg and 20 mg/kg cordycepin treatment groups decreased significantly compared to the control group. PET/CT was used for in vivo imaging of ^18^F-FDG-labeled glucose to observe its metabolism in various parts of the body; the glucose uptake value was expressed as SUVmax. Most of the studies have shown that tumors have a high uptake of ^18^F-FDG. The white arrow indicates the location of the tumor. The current results showed that the SUVmax of the 10 mg/kg and 20 mg/kg cordycepin treatment groups was significantly lower than that of the control group (Figure 5(b)), suggesting that cordycepin reduces glucose uptake in tumor-bearing nude mice. TUNEL staining was used to detect the apoptosis of tumor tissues. The results showed that the number of apoptotic bodies increased significantly in the 10 mg/kg and 20 mg/kg cordycepin treatment groups (Figure 5(c)). We also found that the combination treatment was more effective than cordycepin and 5 FU alone. The paraffin sections of tumor tissues of nude mice were stained with IHC to detect the expression of HK2, PKM2, and LDHA, the key enzymes of aerobic glycolysis. The results showed that, compared to the control group, the protein expression levels of HK2, LDHA, and PKM2 in the cordycepin treatment group were decreased (^∗^*P* < 0.05 or ^∗^*P* < 0.01; Figure 5(d)). This phenomenon suggests that cordycepin regulates the expression of key enzymes of aerobic glycolysis metabolism in HCC. Some tumor tissues from nude mice were randomly selected in each group, and the expression of HK2, PKM2, and LDHA proteins in tumor tissues was detected by Western blotting. The results showed that, compared to the control group, the expression of HK2 was downregulated in the 10 mg/kg and 20 mg/kg cordycepin groups, and the expression of PKM2 and LDHA was significantly downregulated in the 20 mg/kg cordycepin group (^∗^*P* < 0.05 or ^*∗∗*^*P* < 0.01; Figure 5(e)). Therefore, we verified that cordycepin suppresses tumor growth in vivo by regulating aerobic glycolysis. According to the results, cordycepin inhibits tumor growth by mediating aerobic glycolysis, as shown in Figure 6.

## 4. Discussion

Liver cancer results in almost one million deaths annually worldwide, most of which are associated with metastasis [25]. The treatment of liver cancer is predominantly based on surgical resection combined with radio-, chemo-, and targeted-therapy, which inhibits the malignant proliferation of liver cancer cells [26].

Normal cells undergo the four processes of proliferation, senescence, apoptosis, and death. For normal cells, senescence and apoptosis belong to the cell fault protection program, which is conducive to a functional life. Tumor cells are different from normal cells as they suppress cell fault protection and hyperproliferate. Therefore, the proliferative activity of tumor cells is a critical prognostic index for the diagnosis of cancer, and the measurement of cell proliferation can provide valuable information for the prognosis of patients [27].

The Warburg effect suggests that tumor cells provide energy and substances for their own growth through aerobic glycolysis. This is the metabolic characteristic of most tumors [28], and the feature provides a direction for tumor treatment. Glucose is not only the substance needed by the human body for survival but also the most important source of energy. Thus, reducing glucose uptake and inhibiting the aerobic glycolysis pathway can indirectly inhibit tumors. Combining the current results and the relevant literature, the detection of glucose metabolites in this study revealed that cordycepin affects the expression level of glucose metabolites in cells after administration. Thus, it is speculated that the effect of cordycepin on HCC cells might be mediated through the glycolysis pathway; the specific mechanism needs to be further explored.

Cordycepin is considered a natural agonist of AMPK, a key regulator of energy metabolism and mitochondrial diagenesis [29]. Moreover, AMPK has attracted wide attention as a potential therapeutic target for metabolic diseases, including type 2 diabetes and cancer [30]. Xx et al. demonstrated a significant protective effect of cordycepin on mice under conditions of hepatic steatosis, inflammation, liver injury, and metabolic stress by activating the AMPK signaling pathway [31]. Wang et al. found that cordycepin prevents radiation ulcers by inhibiting cell senescence via NRF2 and AMPK in rodents, and activation of AMPK or NRF2 might be the therapeutic targets for preventing cell senescence and radiation ulcer [13]. In various diseases, cordycepin has been shown to activate AMPK and regulate the downstream metabolic pathways. Therefore, it is a promising natural agonist of AMPK. Based on the finding that cordycepin regulates glucose metabolism in liver cancer cells, this study verified that cordycepin regulates the glucose metabolism pathway in tumor cells through phosphorylation of AMPK and downstream Akt. The specific target molecules of the glucose metabolism pathway have not been explored in this study, which is essential in the future. In vivo studies demonstrated that the combination of cordycepin and 5 FU had a better anticancer effect than either of the drugs alone, which provided directions for follow-up studies.

The in vitro and in vivo experiments showed that cordycepin inhibits the proliferation and induces the apoptosis of HCC. In vitro mechanism studies showed that cordycepin inhibits the growth of HCC cells through the AMPK/Akt-HK2/PKM2/LDHA regulatory axis. In conclusion, cordycepin inhibits the growth of liver cancer by regulating the aerobic glycolysis pathway. These findings provide a new mechanism for cordycepin in antitumor therapy and a new research strategy for traditional Chinese medicine and tumor metabolism.

## Data Availability

All remaining data are available within the article and supplementary files or available from the authors upon request.

## Supplementary Materials

Supplementary Materials Supplementary materials include materials and methods (electron microscopy), one figure, and two tables. Supplementary Figure 1 showed that cordycepin does not induce the autophagy cell death pathway. The antibody information used in this study is listed in Supplementary Table 1. The primer sequences in this study are listed in Supplementary Table 2.
