# Supplementary material for: Cordycepin Inhibits the Growth of Hepatocellular Carcinoma by Regulating the Pathway of Aerobic Glycolysis
Source: Evid Based Complement Alternat Med. 2022 Nov 23;2022:6454482. doi: 10.1155/2022/6454482 (PMC9711956; doi:10.1155/2022/6454482)
Supplement: Supplementary Materials — include materials and methods (electron microscopy), one figure, and two tables. Supplementary Figure 1 showed that cordycepin does not induce the autophagy cell death pathway. The antibody information used in this study is listed in Supplementary Table 1. The primer sequences in this study are listed in Supplementary Table 2. [file 6454482.f1.docx]

**Supplementary Material**

**Materials and Methods**

**Electron Microscopy**

Collect cells precipitation after centrifuge, requiring the precipitation should be at least mung beans size. The TEM fixative was added to the tube and let the precipitation re-suspended in the fixative, and then fixed at 4℃ for preservation and transportation.The fixed cells were centrifuged. The 0.1 M PB (pH 7.4) was added into the tube after supernatant was discarded, and then the precipitation was re-suspended and washed in PB for 3min. This washing step was repeated for 3 times. The other Processing of samples were finished by wuhan servicebio technology CO.,LTD.The samples were examined with a HITACHI 7650 electron microscope operated at 100 Kv. For quantitative analysis of autophagy, the number of autophagosome per viable cell was scored. For quantification of viable cells using electron micrographs of 10 single cells from multiple distinct from each specimen.

**
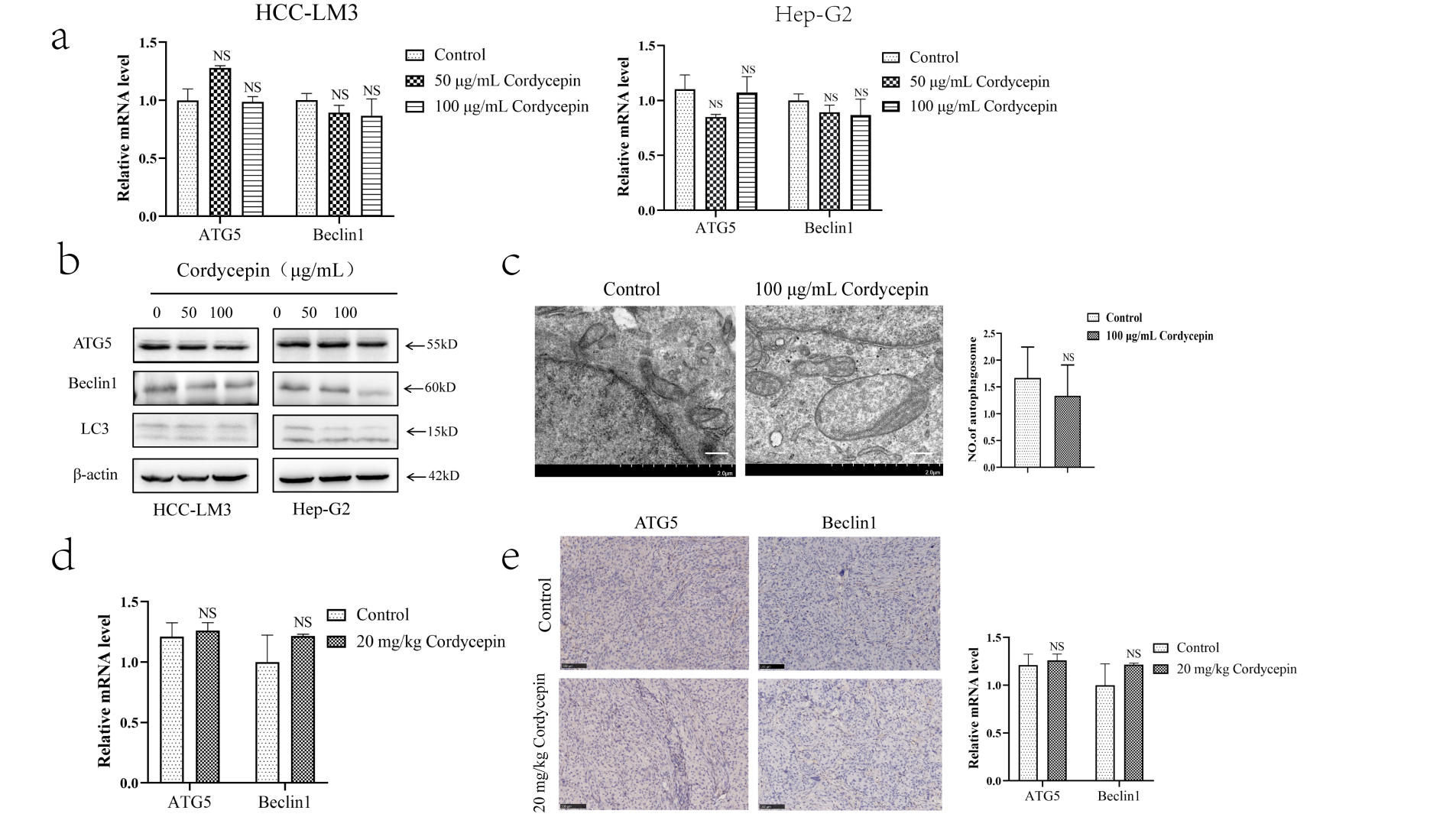
**

**Supplementary figure1. Cordycepin does not induce autophagy cell death pathway. (**a) The gene expression of ATG5 and Beclin1 in HCC-LM3 and Hep-G2 cells.(b) The protein expression of ATG5、Beclin1 and LC3 in HCC-LM3 and Hep-G2.(c)The number of autophagosomes in the control group and 100 μg/mL cordycepin group.(d)The relative mRNA level of ATG5 and Beclin1 of tumor tissues in HCC-LM3 tumor-bearing nude mice. (e) IHC staining and statistical analysis of tumor tissues in HCC-LM3 tumor-bearing nude mice and quantification of the expression of ATG5 and Beclin1.

**Supplementary Table1. Antibodies used in this study**

| Regeant | Company | Product code | Dilution rate |
| --- | --- | --- | --- |
| Bcl-2 rabbit Polyclonal Antibody | Beyotime | AF6285 | 1:1000 |
| Caspase-3(active)Rabbit mAb | Beyotime | AC033 | 1:1000 |
| LDHA | Cell signaling technology | 3582S | 1:1000 |
| PKM2 | Cell signaling technology | 4053S | 1:1000 |
| Hexokinase2 Polyclonal Antibody | Proteintech | 22029-1-AP | 1:1000 |
| P-AMPKalpha(T172) Rabbit Ab | Cell signaling technology | 2531S | 1:1000 |
| AMPKalpha Antibody | Cell signaling technology | 2532S | 1:1000 |
| Akt Rabbit Antibody | Cell signaling technology | 9272S | 1:1000 |
| p-Akt Rabbit mAb | Cell signaling technology | 4058S | 1:1000 |
| HRP labeled goat resistant mouse IgG | EpiZyme | LF101 | 1:10000 |
| HRP labeled goat resistant rabbit | Beyotime | A0208 | 1:10000 |
| LC3 | Cell signaling technology | 12741 | 1:1000 |
| ATG5 | Cell signaling technology | 12994 | 1:1000 |
| Beclin1 | Cell signaling technology | 3495 | 1:1000 |

**Supplementary Table 2. Primers used in this study**

| **Primers for Real-time PCR** | | |
| --- | --- | --- |
| **Protein** | | **Sequence (5’→3’)** |
| Cleaved Caspase3  Bcl-2 | F  R  F  R | ACTGATGAGGAGATGGCTTGC  GGACTGGATGAACCACGACC  CAGGTGGAGGCAAATCTTCGT  ACCCTGTTAATCCGTTCGTTTT |
| AMPK | F | GGGAAAGTGAAGGTGGGCAA |
|  | R | GATGTGAGGGTGCCTGAACA |
| ACC  FASN | F  R  F  R | ATGTCTGGCTTGCACCTAGTA  CCCCAAAGCGAGTAACAAATTCT  AAGGACCTGTCTAGGTTTGATGC  TGGCTTCATAGGTGACTTCCA |
| CB | F | AGATGTAGGCCGGGTGATCT |
|  | R | CCGCCCTGGATCATGAAGTC |
| HK2 | F | AGCCCTTTCTCCATCTCCTT |
|  | R | GCTTGCCTACTTCTTCACGG |
| PKM2  G6PD | F  R  F  R | ATGTCGAAGCCCCATAGTGAA  TGGGTGGTGAATCAATGTCCA  CGCCTCACAGTGGCTGACATC  ACCCCAGGTGGAGGGCATT |
| LDHA | F  R | AACTTGGCGCTCTACTTGCT  GGACTTTGAATCTTTTGAGACCTT |
| IDH1 | F | TCCATTAAGCAATCCAGCCGA |
|  | R | CCCAGATACCATCAGACTGAGC |
| AKT | F | GCCGCCTGATCAAGTTCTCC |
|  | R | TTCAGATGATCCATGCGGGG |
| PFKFB2 | F | GAGGCTAGAACAGGAAGTTA |
|  | R | CACATTAGGCAGATCTCCAG |
| ATG5 | F | TGTGCTTCGAGATGTGTGGTT |
|  | R | GTCAAATAGCTGACTCTTGGCAA |
| Beclin1 | F | ATGGAGGGGTCTAAGGCGTC |
|  | R | TCCTCTCCTGAGTTAGCCTCT |
